# Supplementary material for: Interactions between two QTLs for time to anthesis on spike development and fertility in wheat
Source: Sci Rep. 2021 Jan 28;11:2451. doi: 10.1038/s41598-021-81857-6 (PMC7843729; doi:10.1038/s41598-021-81857-6)
Supplement: Supplementary file 2 — Supplementary Information 2. [file 41598_2021_81857_MOESM2_ESM.docx]

**Interactions between two QTLs for time to anthesis on spike development and fertility in wheat**

Priyanka A. Basavaraddi^1^, Roxana Savin^1^, Luzie U Wingen^2^, Stefano Bencivenga^2^, Alexandra M. Przewieslik-Allen^3^, Simon Griffiths^2^, Gustavo A. Slafer^1,4*^

1 Department of Crop and Forest Sciences, University of Lleida - AGROTECNIO Center, Av. R. Roure 191, 25198 Lleida, Spain

2John Innes Centre, Norwich Research Park, Colney Ln, Norwich, NR4 7UH, United Kingdom

3 University of Bristol, Life Sciences Building, 24 Tyndall Avenue, Bristol BS8 1TQ

4 ICREA, Catalonian Institution for Research and Advanced Studies, Spain

* Corresponding author. Email address: [gustavo.slafer@udl.cat](mailto:gustavo.slafer@udl.cat)

**-Supplementary Figures (7 supplementary Figs)**

**-Supplementary Material** (“Supplementary material_ParagonxBaj_genotypes and map.xlsx”): Genotype data (35k Axiom Wheat Breeders’ array) and genetic map (MSTmap) of the Paragon x Baj population.

Supplementary Figure S1. Heading date QTL on chromosome 2B, 2D and 7D. LOD scores are plotted along the chromosome axis. Marker names and position are listed underneath, along the chromosome axis. The peak marker is highlighted in red and the markers bordering the confidence interval in black. The extend of the confidence interval is shown as horizontal blue lines underneath the marker names.

Supplementary Figure S2. Dynamics of floret development (dimensionless floret score) in F2, F3, F4 and F5 florets at apical (top panel: a-d), central (middle panel: e-h) and basal (bottom panel: i-l) positions of spike with thermal time from sowing in lines with *Eps-7D-late* (open symbol) and early (closed symbol) allele with the late allele of *Eps-2B* in the background in second cropping season.

Supplementary Figure S3. Dynamics of floret development (dimensionless floret score) in F2, F3, F4 and F5 florets at apical (top panel: a-d), central (middle panel: e-h) and basal (bottom panel: i-l) positions of spike with thermal time from sowing in lines with *Eps-7D-late* (open symbol) and early (closed symbol) allele with the early allele of *Eps-2B* in the background in second cropping season.

Supplementary Figure S4. Dynamics of floret development (dimensionless floret score) in F2, F3, F4 and F5 florets at apical (top panel: a-d), central (middle panel: e-h) and basal (bottom panel: i-l) positions of spike with thermal time from sowing in lines with *Eps-2B-late* (triangles) and *early* (circles) allele with the *late* allele of *Eps-7D* in the background in second cropping season.

Supplementary Figure S5. Dynamics of floret development (dimensionless floret score) in F2, F3, F4 and F5 florets at apical (top panel: a-d), central (middle panel: e-h) and basal (bottom panel: i-l) positions of spike with thermal time from sowing in lines with *Eps-2B-late* (triangles) and *early* (circles) allele with the *early* allele of *Eps-7D* in the background in second cropping season.

Supplementary Figure S6. Number of living floret primordia at apical (top panels: a-d) central (middle panel: e-h) and basal spikelet (bottom panel: i-l) and thermal time from sowing as affected by *Eps-7D* (left panels: a, b, e, f, i and j) and *Eps-2B* genes (right panels: c, d, g, h, k and i) on backgrounds contrasting in the allelic form of the other *Eps* gene (left and right panels within each *Eps* gene) in first cropping season.

Supplementary Figure S7. Number of living floret primordia at apical (top panels: a-d) central (middle panel: e-h) and basal spikelet (bottom panel: i-l) and thermal time from sowing as affected by *Eps-7D* (left panels: a, b, e, f, i and j) and *Eps-2B* genes (right panels: c, d, g, h, k and i) on backgrounds contrasting in the allelic form of the other *Eps* gene (left and right panels within each *Eps* gene) in second cropping season.
